# Spectroscopic fingerprinting of extracellular vesicles from diverse cellular origins by ATR-FTIR for vibrational biomarkers of vector–host interactions

**DOI:** 10.1038/s41598-026-44338-2

**Published:** 2026-03-16

**Authors:** Emine Billur Sevinis Ozbulut, Kenta Hoshino, Yoshitomo Furushima, Yuki Yoshida, Takashi Yamamoto, Hanna Reßin, Shreyans Chatterjee, Jan Münch, Boris Mizaikoff, Rüdiger M. Groß, Lorena Diaz de Leon Martinez

**Affiliations:** 1 Toray Automotive Center Europe, Toray Industries Europe GmbH, Am Gfild 6, Neufahrn bei Freising, 85375 Neu-Isenburg, Germany; 2 Toray Research Center Inc, Shiga, 520-8567 Japan; 3 Institute of Analytical and Bioanalytical Chemistry, Ulm University, Albert- Einstein-Allee 11, 89081 Ulm, Germany; 4 Institute of Molecular Virology, Ulm University Medical Center, Meyerhofstrasse 1, 89081 Ulm, Germany

**Keywords:** Extracellular Vesicles, Attenuated total reflectance (ATR), Fourier transform infrared (FTIR), Chemometrics, Arbovirus transmission, Biochemistry, Biological techniques, Biotechnology

## Abstract

Extracellular vesicles (EVs) are nanoscale lipid bilayer structures that facilitate intercellular communication across biological systems. Although extensively studied in mammals, their spectral and biochemical characteristics in invertebrate hosts relevant to viral transmission remain poorly understood. In this study, Attenuated Total Reflectance–Fourier Transform Infrared (ATR-FTIR) spectroscopy was used to characterize and discriminate EVs derived from human dermal fibroblasts (FibEVs), hepatocytes (HepEVs), and *Aedes albopictus* mosquito cells (MosqEVs), alongside synthetic EV-like vesicles (SynEVs). Spectral data were analyzed using Principal Component Analysis (PCA), Canonical Analysis of Principal Coordinates (CAP), and sparse Partial Least Squares–Discriminant Analysis (sPLS-DA). PCA revealed clear clustering according to EV origin, while CAP showed strong group separation (R² ≈ 0.99), with the first two canonical axes explaining 89% of total variance. sPLS-DA identified discriminant wavenumbers associated with lipids, proteins, and nucleic acids, achieving 93% classification accuracy and an average AUC of 0.99. MosqEVs displayed lipid-enriched spectral profiles consistent with insect membrane composition, whereas mammalian EVs were protein-dominant. These findings demonstrate that ATR-FTIR is a rapid, label-free approach for EV discrimination and provide the first vibrational characterization of mosquito-derived EVs, supporting future applications in vector biology, infection surveillance, and exosome quality assessment.

**Supplementary Information:**

The online version contains supplementary material available at 10.1038/s41598-026-44338-2.

## Introduction

Extracellular vesicles (EVs) represent a heterogeneous population of lipid membrane–bound nanoparticles that play crucial roles in intercellular communication and physiological regulation across both prokaryotic and eukaryotic organisms^1^. In the biomedical field, EVs have emerged as valuable diagnostic biomarkers and as vehicles for targeted therapies^2,3^, although most research has focused on mammalian models. However, EVs are secreted by all cells, and e.g., insect cell-derived EVs also play an important role in arbovirus transmission^4–6^.

Current methods for analyzing EVs primarily rely on biophysical characterization via electron microscopy or nanoparticle tracking analysis and compositional analysis using qPCR, Western Blot or corresponding omics techniques. ^7^Although especially the latter methods offer high resolution, they are costly, labor-intensive, and impractical for high-throughput or routine screening studies. In this regard, Attenuated Total Reflectance–Fourier Transform Infrared (ATR-FTIR) spectroscopy has emerged as an innovative methodological alternative. It has already been used to discriminate between EV subpopulations based on the protein-to-lipid spectral ratio^8^ and to detect structural changes associated with pathological conditions^9–11^ as well as a tool for quality control assessment after isolation^12^. The simplicity of sample preparation, label-free detection and the ability to analyze global molecular composition make ATR-FTIR an attractive platform for comparative and diagnostic explorations, including preliminary applications in neurodegeneration^13^ and cancer research^9^. However, its use in studying vesicles from diverse sources, e.g. for in the context host–pathogen interaction is poorly explored.

In this study, different populations of extracellular vesicles representative of both ends of the arbovirus vector-host transmission were selected. EVs obtained from three cell types were analyzed: human dermal fibroblasts (HDFs), hepatocytes (Huh7, a relevant model for flavivirus infection), and C6/36 (*Aedes albopictus)* mosquito cells. These vesicles are of similar nanoscale diameters, yet differ in composition, reflecting their cells of origin. For example, insect cell derived vesicles exhibit greater deformability due to highly symmetrical membranes. At the same time, fibroblast-derived EVs are rich in adhesion molecules^14,15^, while hepatocyte derived vesicles are enriched in components of lipid-synthesis pathways^16^.

In this work, we propose ATR-FTIR spectroscopy as an innovative tool for the spectral and compositional discrimination of EVs derived from various sources. The central hypothesis is that the infrared spectra of EVs reflect their composition, derived from their biological origin, and can functionally differentiate vesicles involved in viral transmission or other diseases. This study presents the first ATR-FTIR spectral characterization of mosquito-derived EVs, providing a methodological foundation for new strategies in infectious disease surveillance and monitoring through rapid, label-free vibrational analysis.

## Materials and methods

### Cell cultures

EVs were isolated from three different cell systems representing both vertebrate and invertebrate origins. Huh7 cells were cultured in Dulbecco’s Modified Eagle Medium (DMEM) supplemented with 10% fetal calf serum (FCS), L-glutamine, and penicillin–streptomycin (PenStrep) under standard conditions (37 °C, 5% CO₂). After expansion to confluence in a 10-layer Cell Factory, cultures were switched to serum-free OptiMEM medium containing PenStrep, L-glutamine, non-essential amino acids (NEAA), and sodium pyruvate for vesicle production. HDF cells were grown in a Cell Factory (6320 cm² growth area) using serum-free Gibco OptiMEM supplemented with PenStrep and L-glutamine and harvested after four days of culture at 92% viability. Similarly, *Aedes albopictus* C6/36 cells were maintained under insect cell conditions (28 °C, without CO₂) in serum-free Gibco OptiMEM supplemented with PenStrep, GlutaMAX, NEAA, sodium pyruvate, and HEPES, and their supernatant was collected after four days of culture with 95% cell viability. Primary HDF cells were obtained from CELLnTEC (CnT Dermal Fibroblasts, Juvenile, Single Donor); C6/36 cells (ATCC CRL-1660) were provided by the Institute for Virology, Charité, Berlin, Germany, and Huh7 cells were provided by the Department for General and Visceral Surgery, Ulm University, Ulm, Germany. None of the cell lines were obtained from patients.

### EV isolation and purification

EV purification was performed as previously described^17^. Conditioned media from all cultures were first centrifuged to remove cellular debris and filtered through a 0.45 μm membrane. The clarified supernatants were then concentrated and pre-purified by tangential flow filtration (TFF) using a 300 kDa molecular weight cut-off (MWCO) membrane. Further purification was achieved through bind-elute size exclusion chromatography (BE-SEC) employing CaptoCore 700 resin, and the EV-containing fractions were concentrated using 100 kDa MWCO ultrafiltration spin filters to obtain the final purified preparations. Purified EVs were resuspended in PBS-HAT buffer^18^. Samples were stored at − 80 °C for long-term preservation and maintained at 4 °C for up to four weeks after thawing. Re-freezing was avoided to prevent vesicle aggregation and alterations in size distribution.

### Synthesis of EV-like particles

Synthetic EV-like vesicles (SynEVs) were prepared from defined lipid mixtures to mimic the composition and biophysical properties of biological EVs. Lipid components (all from Avanti Polar Lipids) included phosphatidylcholine (18:1, 24 mol%), phosphatidylserine (18:1, 10 mol%), phosphatidylethanolamine (18:1, 5 mol%), cholesterol (45 mol%, from ovine wool), sphingomyelin (18:1, 15 mol%), and Top-Fluor–phosphatidylcholine (18:1, 1 mol%), totaling a final lipid concentration of 3 mM. Lipids were dissolved in chloroform and evaporated under an inert gas stream to form a thin lipid film. The resulting lipid cake was rehydrated in PBS containing 0.1 mg/mL human serum albumin (Albutein^®^, Grifols) and a 500 nM synthetic 35-mer oligonucleotide (Biomers).

The rehydrated lipid suspension was subjected to 21 sequential extrusions through 100 nm polycarbonate membranes to generate unilamellar, monodisperse vesicles. To simulate membrane-associated proteins, bovine lactadherin (4 µg/mL) was added post-extrusion. The resulting vesicle preparation was resuspended in PBS-HAT buffer. Samples were stored at − 80 °C for long-term preservation and maintained at 4 °C for up to four weeks after thawing. Re-freezing was avoided to prevent vesicle aggregation and alteration of size distribution.

### EV Characterization

The concentration and size distribution of EVs derived from HDF, Huh7, and C6/36 cells were determined by Nanoparticle Tracking Analysis (NTA) using the ZetaView instrument (Particle Metrix GmbH). Samples were diluted in PBS to achieve an optimal measurement range of 100–300 particles per frame, and triplicate measurements were collected for each EV preparation.

EV surface marker profiling was performed using the Miltenyi Biotec MACSplex EV IO Kit (#130-108-813) combined with Alexa Fluor 647–conjugated bovine lactadherin (CellSystems) for phosphatidylserine (PS) detection as described^17^. Samples were incubated with capture beads overnight according to the manufacturer’s instructions, followed by dual staining with tetraspanin antibodies (CD9, CD63, CD81) and lactadherin. Fluorescence signals were acquired on a CytoFLEX flow cytometer (Beckman Coulter), and data were analyzed using CytExpert software. All measurements were performed in duplicate, blanks were subtracted, and averaged values were used for subsequent analysis.

EVs were prepared for transmission electron microscopy (TEM) as previously described elsewhere^19^. For that, 5 µL of the respective sample suspension was left to adhere onto a formvar and carbon-coated 200 mesh copper grid for 1 min. All steps were performed at room temperature. The grid was then washed three times with aqua bidest, stained with 2% (w/v) uranyl acetate solution in aqua bidest, and then air dried. Finally, the dried grid was examined with a JEM-1400 transmission electron microscope (JEOL, Tokyo, Japan) at 120 kV and images were acquired with a CCD camera (Veleta, Olympus, Tokyo). Imaging conditions were optimized to maintain extracellular vesicle ultrastructure during preparation while enabling high-resolution visualization of vesicle size distribution, bilayer integrity, and nanoscale interactions.

### ATR-FTIR Analysis

Prior to ATR-FTIR analysis, all EV solutions were normalized by particle concentration to minimize variability in spectral intensity arising from differences in sample load. All EV suspensions were normalized to a concentration of approximately 10^10^ particles/mL, corresponding to the least concentrated biological EV preparation as determined by nanoparticle tracking analysis. This normalization strategy ensured identical particle-loading conditions across all experimental groups without artificially concentrating low-yield samples, while providing sufficient infrared absorbance within the linear response range of the ATR-FTIR setup. To account for potential background contributions, PBS-HAT buffer alone was analyzed separately under identical conditions to characterize its intrinsic spectral profile and evaluate the need for baseline correction or spectral subtraction. The resulting spectra showed that the PBS-HAT signal was negligible compared to that of the EV-containing samples, confirming that buffer components did not significantly interfere with the EV spectral features (**Figure S5**).

Infrared spectra of EV solutions were recorded in the mid-infrared region (4000 –400 cm^− 1^) using an Alpha II spectrometer (Bruker Optics GmbH, Ettlingen, Germany) equipped with a platinum ATR module featuring a monolithic diamond single-reflection crystal, a CenterGlo™ infrared source, and a temperature-stabilized DTGS detector. Instrument control and spectral processing were performed using OPUS version 8.8.4 (Bruker Optics GmbH). For each measurement, 2 µL of the EV suspension was carefully deposited onto the ATR crystal using a calibrated micropipette to ensure consistent sample loading. Afterwards samples were air-dried at room temperature using a gentle manually intermittent air stream applied from a distance of approximately 15–20 cm. Airflow was applied in short pulses to facilitate uniform evaporation while minimizing sample displacement. Complete drying was typically achieved approximately in 10 min. Uniform coverage of the ATR crystal was verified by visual inspection, ensuring homogeneous film formation without visible aggregation, edge thickening, or uncovered regions. Spectra were obtained by co-adding 64 scans at a spectral resolution of 2 cm^− 1^, using a background spectrum of air acquired under identical instrumental settings. For each sample, 17 replicates were analyzed.

To prevent cross-contamination, the ATR crystal was thoroughly cleaned between measurements with deionized water, isopropanol, and methanol, followed by gentle wiping with optical paper until the baseline signal was restored. The order of sample acquisition was randomized to minimize systematic bias. For each biological condition, spectral and sample replicates from the same EV group were recorded consecutively to avoid repeated freeze–thaw cycles that could alter vesicle composition or integrity.

Prior to data analysis, all spectra underwent preprocessing to correct baseline variations and instrumental drift. An initial straight-line correction was applied using 203 points across the full spectral range to establish a uniform baseline. Subsequently, a manual refinement of the baseline was performed by adding approximately 15–20 anchor points in specific regions of interest—particularly around 1765, 1717, 1596, 1498, 1296, 890, 816, and 400 cm^− 1^—as well as in spectral zones affected by atmospheric interference. This correction procedure ensured the accurate alignment and normalization of all spectra, minimizing background noise and enhancing the resolution of characteristic absorption bands associated with EV biochemical components. After pre-processing the area of interest from 1800 –800 cm^− 1^ was selected for chemometric analysis.

### Chemometric Data Analysis

Spectral datasets obtained from ATR-FTIR measurements were subjected to chemometric analysis to explore, classify, and discriminate EV samples according to their cellular origin and composition. Prior to statistical modeling, spectral data were auto-scaled (mean-centered and scaled to unit variance) to ensure that all wavenumbers contributed equally to the analysis, thereby minimizing bias caused by differences in signal intensity across spectral regions.

To identify intrinsic trends and visualize global variance among samples, a Principal Component Analysis (PCA) was first applied. PCA served as an unsupervised exploratory tool to reduce data dimensionality while retaining the maximum variance, allowing for the identification of potential outliers, clustering patterns, and similarities among EV spectra. The number of principal components retained was determined using a cumulative variance criterion. Principal components were included until the cumulative explained variance exceeded 80% of the total variance, ensuring adequate representation of spectral variability while minimizing inclusion of noise-dominated components. PCA was additionally employed for outlier detection using Hotelling’s T² statistic and visual inspection of score plots within the 95% confidence ellipse. Samples exceeding Hotelling’s T² critical threshold or located outside the confidence limits were considered potential outliers. For a more discriminant assessment, Canonical Analysis of Principal Coordinates (CAP) was performed to maximize group separation based on prior class information. CAP enabled the evaluation of how well spectral profiles could differentiate EVs derived from various biological origins while providing quantitative measures of model fit and classification accuracy. To identify wavenumbers contributing most to class discrimination, a sparse Partial Least Squares–Discriminant Analysis (sPLS-DA) model was applied. This supervised method allowed simultaneous dimensionality reduction and variable selection, pinpointing the spectral features most relevant to the biochemical differences among EV types while mitigating overfitting through sparsity constraints. In contrast to PCA, sPLS-DA is a supervised method that maximizes covariance between predictor variables and class membership rather than total variance alone. Therefore, the proportion of total variance explained does not directly reflect discriminative performance. The number of components was determined based on cross-validation accuracy and model stability. Finally, the Receiver Operating Characteristic (ROC) curve was generated from the sPLS-DA classification results to evaluate model performance in terms of sensitivity and specificity. The Area Under the Curve (AUC) was calculated as a global measure of discriminatory power, providing an objective assessment of the model’s ability to correctly classify EVs from different cellular and synthetic origins. For ROC computation, the directionality of the class prediction score was verified to ensure correct assignment of the positive class. In cases where an AUC value below 0.5 resulted from reversed score polarity (i.e., anti-correlated ranking of the positive class), the orientation of the prediction score was inverted, which is mathematically equivalent to applying AUC corrected = 1 − AUC. This procedure does not alter class separation but ensures correct ROC interpretation. Data processing and statistical analyses were performed using MetaboAnalyst 6.0 (www.metaboanalyst.ca) and a self-developed Python script for complementary multivariate modeling and visualization.

## Results

### Characterization of EVs

NTA confirmed that all EVs displayed particle sizes and concentrations consistent with typical EV populations (**Figure ****S1**). HDF-derived EVs (FibEVs) showed a median diameter of 148.5 nm with a particle concentration of 5.9 × 10^10^ particles/mL, indicative of a heterogeneous but predominantly exosome-sized population. Huh 7 derived EVs (HepEVs) exhibited a similar size distribution (median ~ 125.33 nm) and a comparable concentration (5.6 × 10^10^ particles/mL). In contrast, mosquito C6/36-derived EVs (MosqEV) were slightly smaller, with a median diameter of 137.4 nm and a notably higher particle concentration of 4.9 × 10^11^ particles/mL. As a synthetic reference, SynEVs presented a narrower size distribution centered at 116.0 nm and the highest overall concentration (7.9 × 10^12^ particles/mL), confirming the efficiency and uniformity of the extrusion-based preparation. We note that these SynEVs serve as a minimalistic, biophysical, and biochemical mimic of EVs, as they are lipid nanoparticles in EV-like size ranges, containing EV-typical lipids, and generic protein and nucleic acid cargo. Analysis of typical EV surface proteins showed classical EV markers CD63 and CD81 on both human cell-derived EVs, although source-specific differences, such as high signals for CD44 on FibEVs and CD133-1 for HepEVs (**Figure S2**) highlight compositional differences.

TEM imaging of negative-stained samples revealed the characteristic cup-shaped morphology and typical size distributions of 50–150 nm diameter for EVs from all origins and synEVs. MosqEVs exhibited a heterogeneous population of rounded, membrane-bound vesicles. The vesicles displayed defined lipid bilayers and small aggregates, suggesting active vesicle secretion typical of insect cells. HepEVs showed a relatively uniform morphology with spherical vesicles displaying smooth membranes and intact structures. FibEVs appeared as discrete, rounded vesicles occasionally forming small clusters. The high structural definition and clear bilayer contrast confirmed the quality and homogeneity of the fibroblast-derived vesicles. In contrast, SynEVs exhibited a more uniform and monodisperse population of vesicles. These artificial vesicles presented smooth, spherical morphologies with consistent electron density, validating the reproducibility of the extrusion-based preparation and confirming their structural similarity to natural EVs (**Figure S3**).

### ATR-FTIR Chemometric Augmented Analysis

**Fig. 1:**
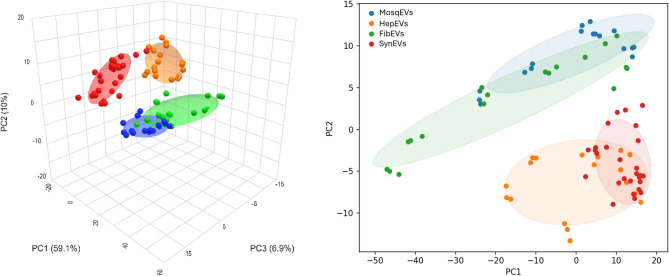
PCA from MosqEVs (blue circle); HepEVs, (orange circles); FibEVs (green circles) and SynEVs (red circles).

With the data from the IR spectra obtained from the EVs analysis, the differences in the spectral fingerprints of each one of the study groups were evaluated and characterized. No statistical outliers were detected based on Hotelling’s T² analysis, and all spectra were retained for further multivariate evaluation. PCA of the IR spectra revealed clear clustering of EVs according to their origin (Fig. 1). The PCA overall variance was 84.04% through 5 PCs (PC1 = 59.14%, PC2 = 9.94%, PC3 = 6.89%, PC4 = 4.51%, PC5 = 3.52%). The first three principal components accounted for most of the spectral variance, demonstrating distinct separation among MosqEVs, HepEVs, FibEVs and SynEVs. PCA plots showed well-defined group distributions with minimal overlap, indicating that the biochemical composition of EVs varies significantly among sources. Interestingly, FibEVs and HepEVs did not cluster closer together amongst each other than between SynEVs and FibEVs, despite the fact that these are both derived from human cells.

**Fig. 2:**
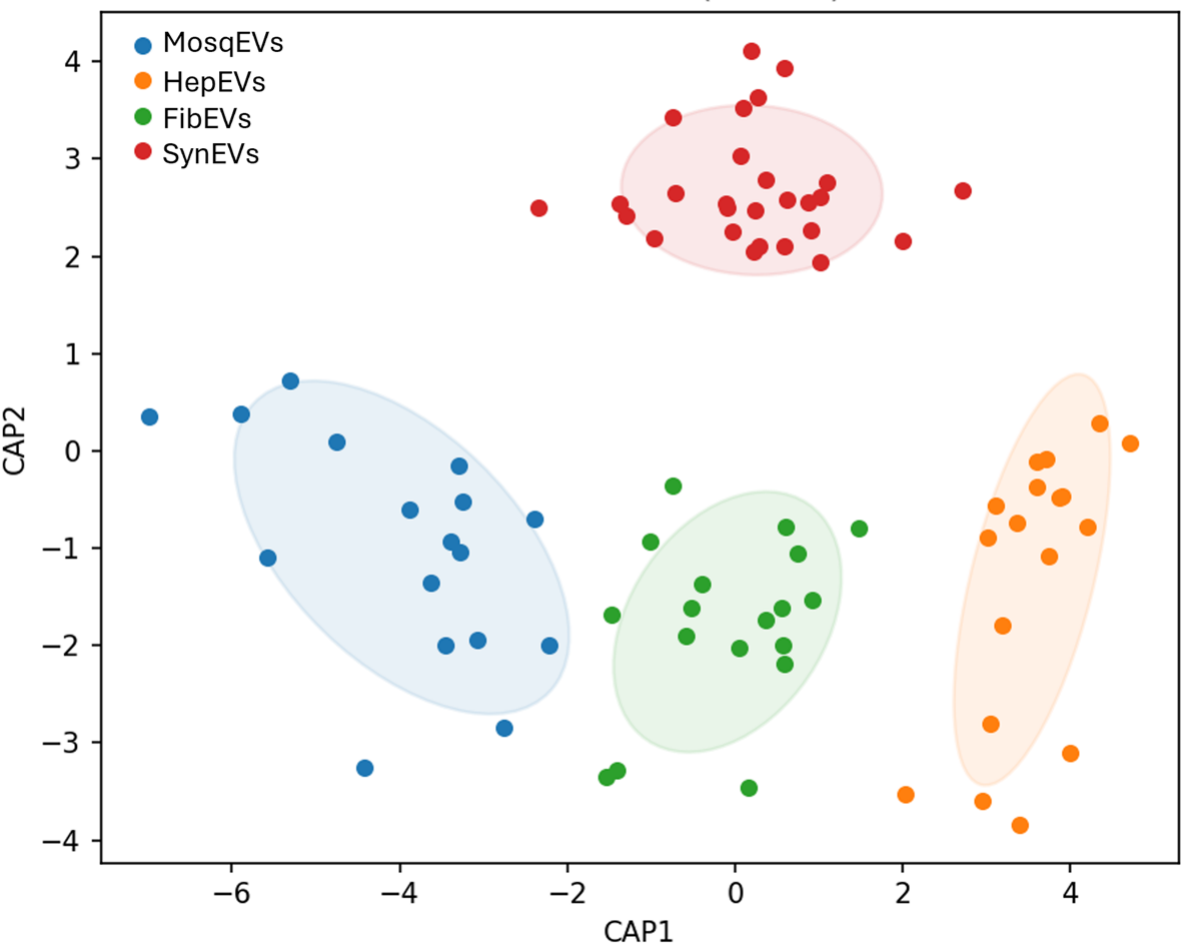
CAP from MosqEVs (blue circles); HepEVs, (orange circles); FibEVs (green circles) and SynEVs (red circles).

**Fig. 3:**
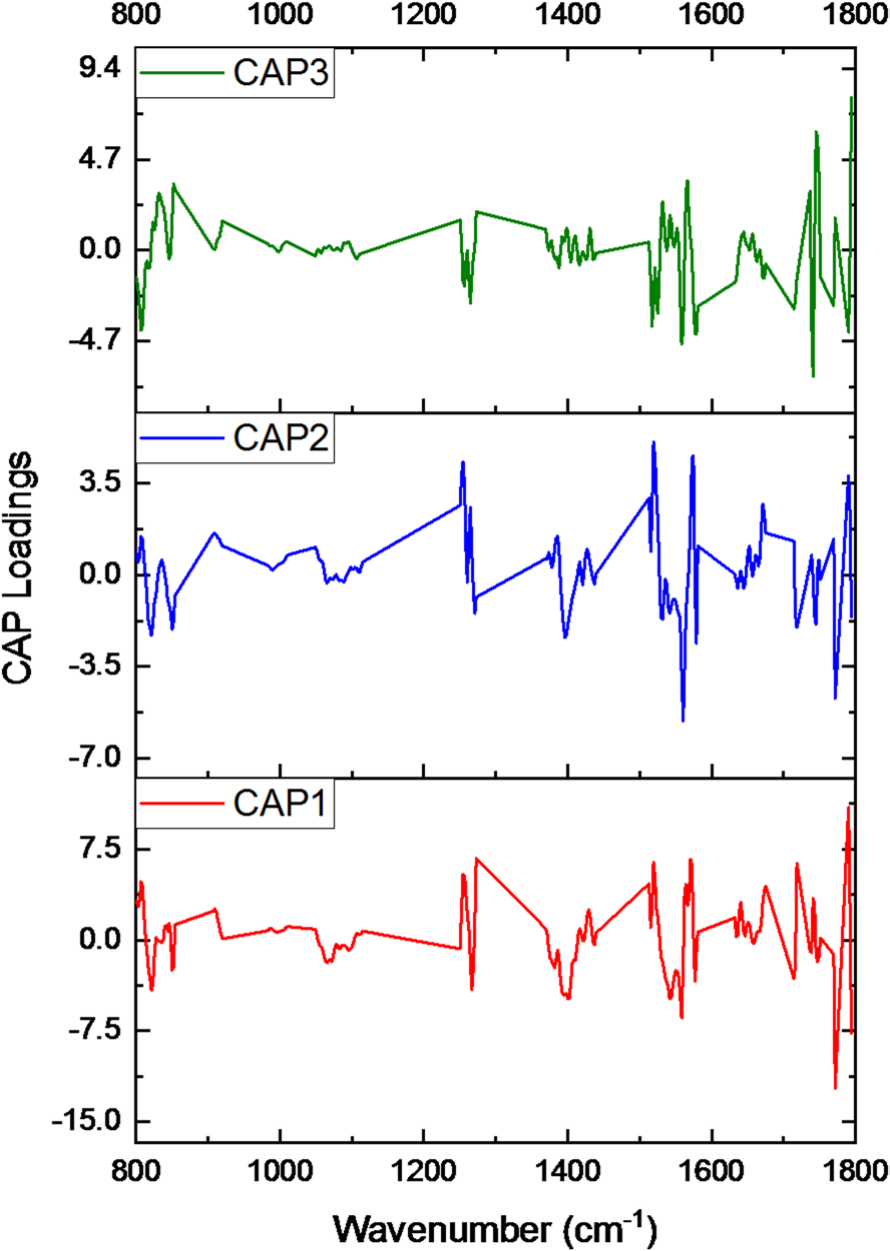
CAP loadings, CAP 1 red, Cap 2 blue, CAP 3 green.

To further assess the discriminatory capabilities of these spectral differences and quantitatively evaluate class separation, a CAP was subsequently performed (Fig. 2). This supervised multivariate method enabled us to maximize the separation between predefined EV groups. CAP based on the Euclidean distance revealed clear group separation, with CAP1 (55.6%) and CAP2 (33.4%) together explaining 89% of the total variance in the IR spectral data (R^2^~ 0.99). CAP1 primarily discriminated MosqEVs, which exhibited negative CAP1 scores, from the other groups with positive values. The further assessment of the CAP loadings revealed the association of each CAP with different vibrations and the regions of interest (Fig. 3; Table 1). This analysis revealed three orthogonal axes that capture complementary biochemical information and support origin-driven discrimination of the vesicle preparations. CAP1 showed a complex fingerprint with major positive loadings at 1790 cm^− 1^ (lipid C = O stretch), 1570–1575 cm^− 1^ (protein amide III/II), 1519–1522 cm^− 1^ (protein/lipid), and 1273 cm^− 1^ (carbohydrate/PO_4_), together with a carbohydrate-ring band at 806 cm^− 1^, indicating concurrent contributions from lipids, proteins, and carbohydrate-rich components. Strong negative loadings on CAP1 at 1771–1794 cm^− 1^ (lipid C = O stretch and ester C = O), 1558 cm^− 1^ (amide II), 1400–1402 cm^− 1^ (COO^–^, carbohydrate), and 1393–1396 cm^− 1^ (carbohydrate) further emphasize that this axis primarily contrasts EV populations according to the balance between esterified lipid carbonyls and carbohydrate-associated vibrations. CAP2 was dominated by protein- and lipid-related features, with positive loadings around 1519–1522 cm^− 1^ (protein/lipid), 1523 cm^− 1^ (protein), 1254 –1252 cm^− 1^ (PO_2_^–^, carbohydrate), and 1790 cm^− 1^ (lipid), while negative loadings at 1560 cm^− 1^ (amide II), 1771 cm^− 1^ (lipid C = O), 1558–1562 cm^− 1^ (amide II) and 1396–1398 cm^− 1^ (COO^–^, carbohydrate) suggest that this axis refines the separation of EVs according to specific protein secondary-structure signatures and associated phospholipid headgroup and carbohydrate modes. In contrast, CAP3 was mainly associated with lipid content but also reflected relevant protein contributions, as indicated by prominent positive bands at 1794 cm^− 1^ (lipid, ester C = O), 1745–1747 cm^− 1^ (lipid ester C = O), 1749 cm^− 1^ (lipid), 1564–1566 cm^− 1^ (protein amide II/III), 851–854 cm^− 1^ (carbohydrate, PO_2_^–^) and 1736 cm^-1^ (lipid ester), together with negative bands at 1741 cm^− 1^ (lipid), 1558 cm^− 1^ (amide II), 1577–1579 cm^− 1^ (amide II), 1790 cm^− 1^ (lipid), 1517 cm^− 1^ (protein/lipid) and 808 cm^− 1^ (carbohydrate ring). Taken together, the CAP loadings indicate that the discrimination of extracellular vesicles of different origins is driven by coordinated changes in esterified and non-esterified lipid carbonyls, protein amide modes, and carbohydrate/phosphate-associated vibrations, supporting the view that origin-specific EV populations exhibit distinct and quantifiable differences in their membrane lipid composition, protein content, and glycosylation patterns.

**Table 1:** CAP loadings peak assignments.

CAP	Top Positive Loadings Peak Assignment (s)	Top Negative Loadings Peak Assignment (s)
CAP1	1790 cm⁻¹ (lipid C = O stretch), 1273 cm⁻¹ (carbohydrate/PO₄), 1570–1575 cm⁻¹ (protein Amide II/III), 1519–1522 cm⁻¹ (protein/lipid), 806 cm⁻¹ (carbohydrate ring)	1771 cm⁻¹ (lipid C = O stretch), 1794 cm⁻¹ (ester C = O), 1558 cm⁻¹ (protein Amide II), 1400–1402 cm⁻¹ (COO–, carbohydrate), 1541–1544 cm⁻¹ (protein Amide II), 1393–1396 cm⁻¹ (carbohydrate)
CAP2	1519–1522 cm⁻¹ (protein/lipid), 1254 cm⁻¹ (PO₂–, carbohydrate), 1790 cm⁻¹ (lipid), 1252–1256 cm⁻¹ (carbohydrate, PO₂–), 1523 cm⁻¹ (protein)	1560 cm⁻¹ (protein Amide II), 1771 cm⁻¹ (lipid C = O), 1558–1562 cm⁻¹ (protein), 1396–1398 cm⁻¹ (COO–, carbohydrate), 819–821 cm⁻¹ (sugar ring)
CAP3	1794 cm⁻¹ (lipid, ester C = O), 1745–1747 cm⁻¹ (lipid, ester C = O), 1749 cm⁻¹ (lipid), 1564–1566 cm⁻¹ (protein Amide II/III), 851–854 cm⁻¹ (carbohydrate ring), 1736 cm⁻¹ (lipid ester)	1741 cm⁻¹ (lipid), 1558 cm⁻¹ (protein Amide II), 1577–1579 cm⁻¹ (protein Amide II), 1790 cm⁻¹ (lipid), 806 cm⁻¹ (carbohydrate), 1517 cm⁻¹ (protein/lipid), 808 cm⁻¹ (carbohydrate ring)

**Fig. 4:**
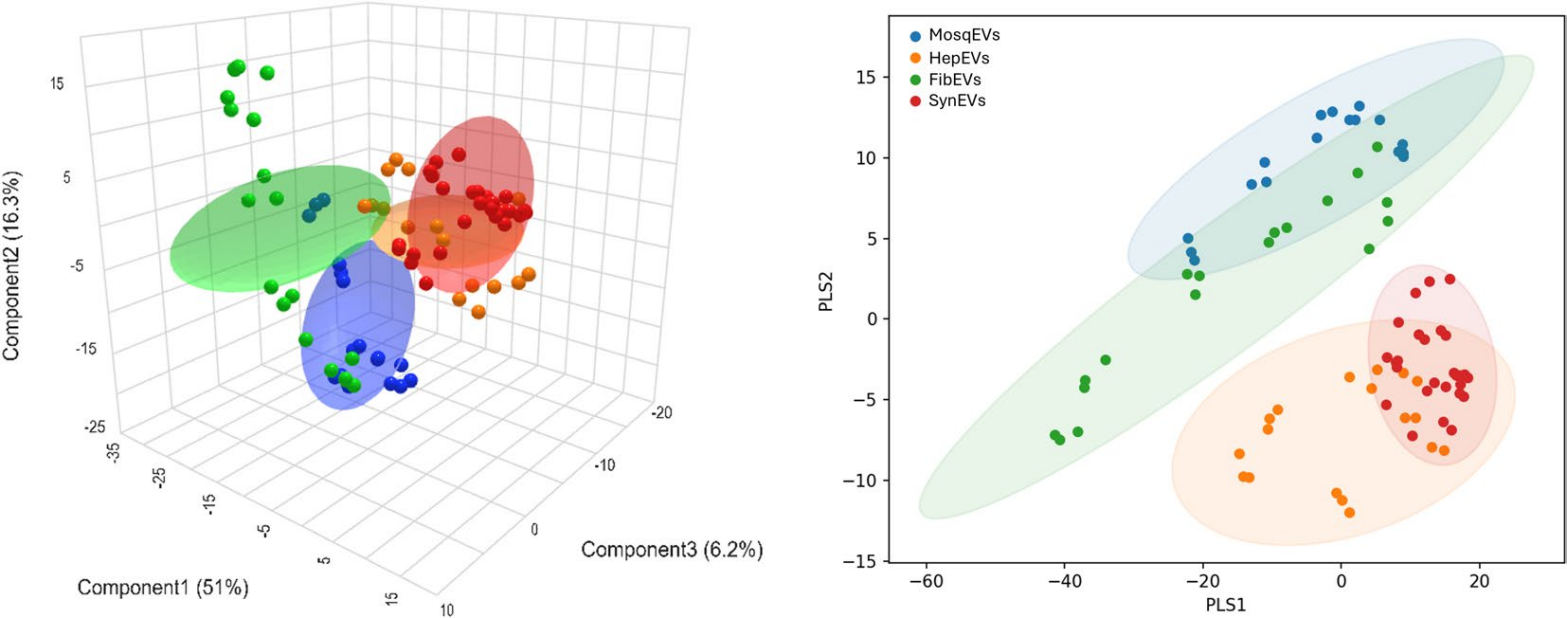
sPLS-DA from MosqEVs (blue circles); HepEVs, (orange circles); FibEVs (green circles), and SynEVs (red circles).

While CAP analysis confirmed clear group separation and strong classification performance, this method does not directly indicate which spectral variables contribute most to the observed discrimination. To address this, a sPLS-DA was conducted (Fig. 4). This supervised technique combines dimensionality reduction with variable selection, allowing the identification of the specific wavenumbers that most strongly differentiate. The first three sPLS components explained 35.2%, 7.1%, and 13% of the total variance and accumulated variance of 77.5% through 5 components, demonstrating high model performance and minimal overlap between clusters. Score plots revealed well-defined separation of the groups. When evaluating model performance, the sPLS-DA presented an error of classification of 6.2, yielding an accuracy of 93% by 63 features. Although the cumulative variance explained by the first three components was 55.3%, this distribution reflects the supervised nature of sPLS-DA, where discriminative information may be distributed across multiple latent variables rather than concentrated in the dominant sources of global variance. Importantly, the model achieved high classification accuracy and AUC values with sparsity constraints limiting the analysis to 63 discriminative features, indicating that classification was driven by biologically meaningful spectral patterns rather than noise.

**Fig. 5:**
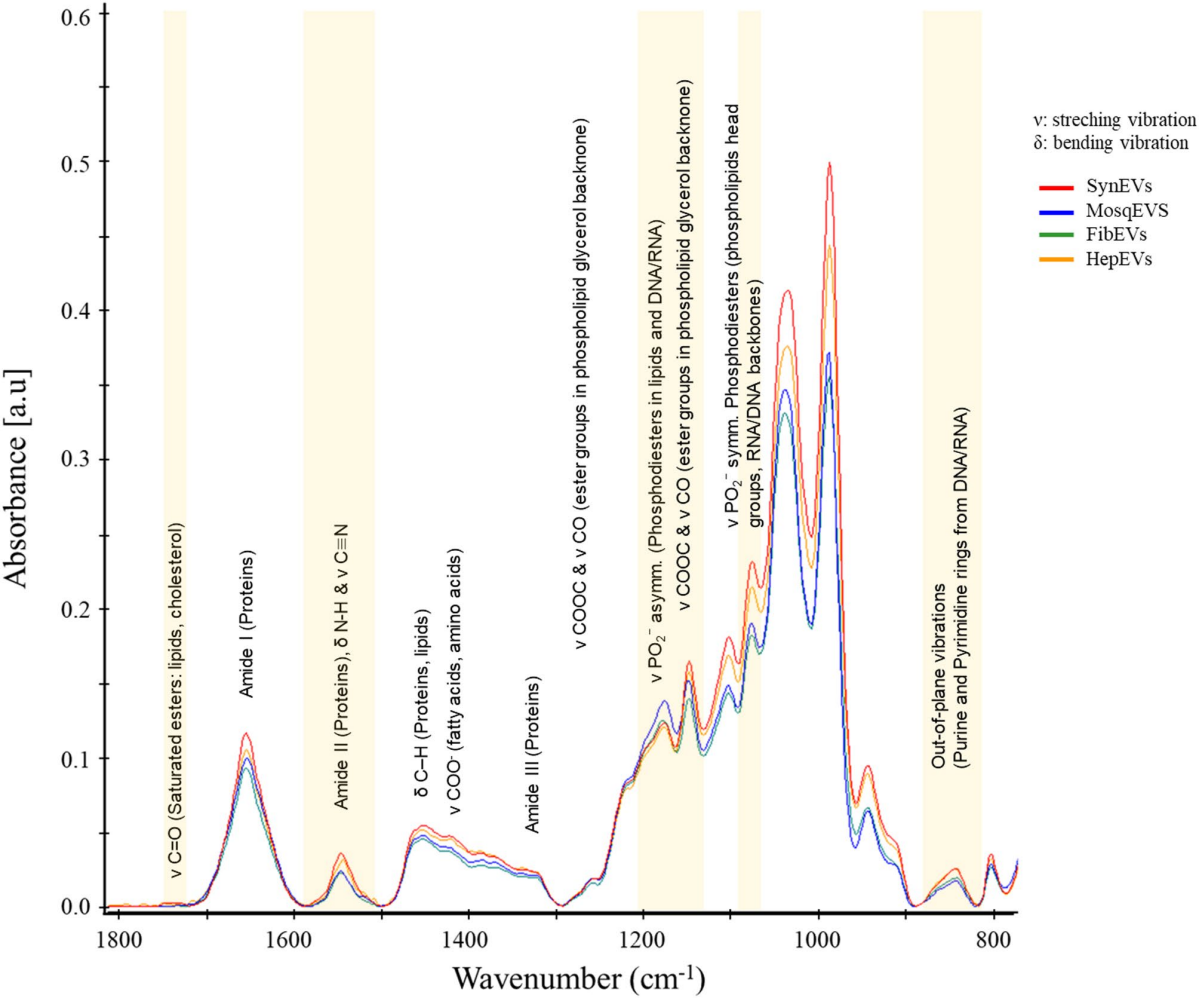
ATR-FTIR Average spectra from each group. Important bands and assignments for group discrimination and sample prediction. MosqEVs (blue), FibEVs (green), HepEVs (orange) and SynEVs (red).

Results from sPLS-DA further confirmed the spectral differentiation among the analyzed EV types. The averaged ATR-FTIR spectra of MosqEVs (blue), FibEVs (green), HepEVs (orange) and SynEVs (red) exhibited distinct absorption patterns across key biochemical regions (Fig. 5). The yellow-highlighted bands indicate the wavenumbers that contributed most strongly to class discrimination, as identified by the sPLS-DA variable importance scores. Specifically, these regions correspond to prominent spectral features: lipid-associated ester carbonyls (ν(C = O), around 1740 cm^− 1^), the protein-related amide II band (N–H bending and C–N stretching, around 1550 cm^− 1^), symmetric stretching of phosphodiesters from RNA/DNA backbones and phospholipid headgroups (around 1080 cm^− 1^), C–O–C and C–O stretching of ester groups and phosphodiesters in lipids and DNA/RNA (1200 –1000 cm^− 1^), and out-of-plane vibrations from purine and pyrimidine rings in nucleic acids (around 900 –800 cm^− 1^). These highlighted regions emphasize the roles of lipid, protein, and nucleic acid vibrational modes in distinguishing different extracellular vesicle types in the dataset depicted. These variations reflect compositional differences in protein secondary structures, lipid chain organization, and nucleic acid content among the EV populations. Consistent with the PCA and CAP analyses, the sPLS-DA model achieved clear discrimination between the three EV groups, reinforcing that each vesicle type possesses a distinct biochemical fingerprint detectable by ATR-FTIR spectroscopy.

**Fig. 6:**
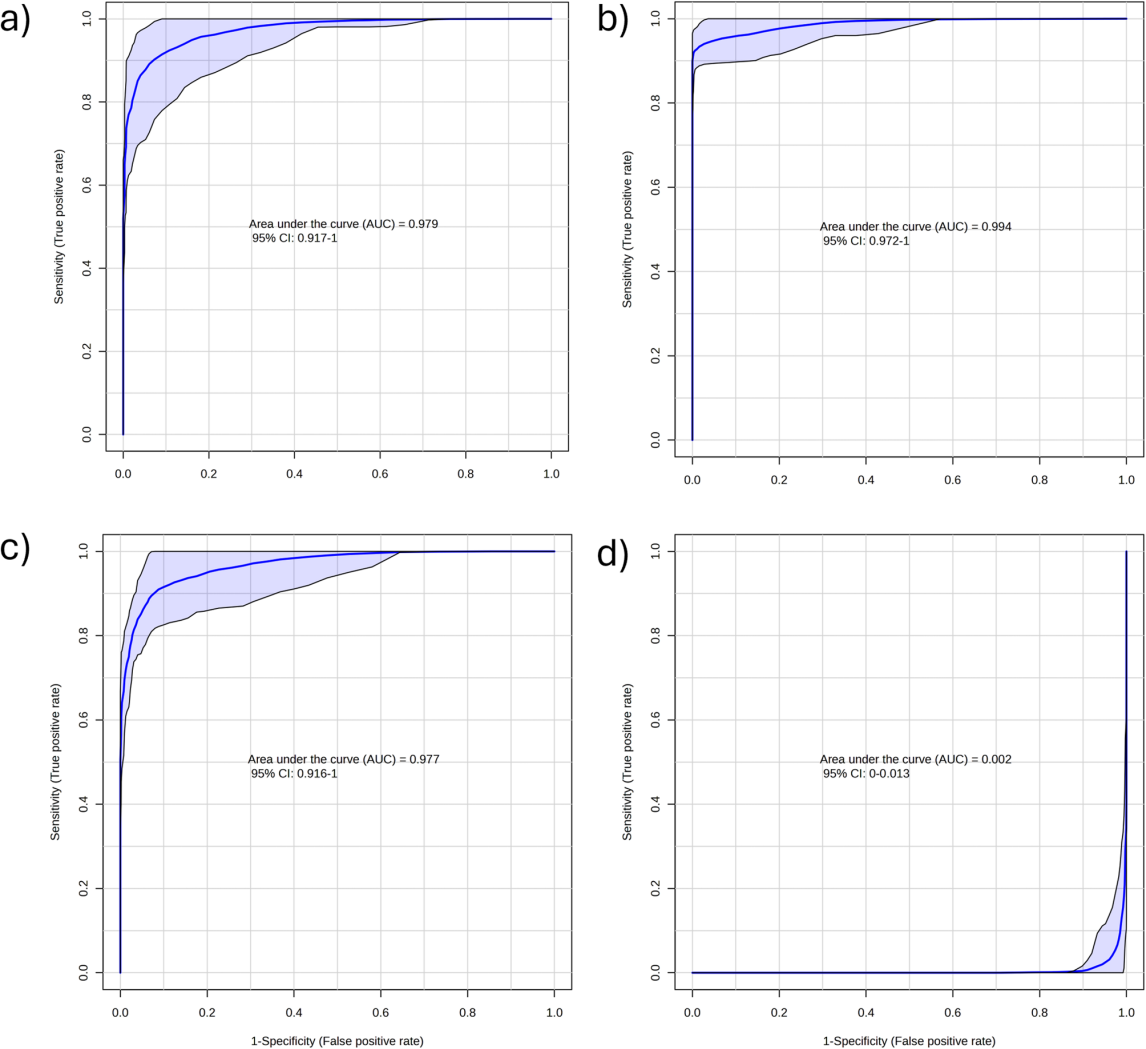
ROC curve for sPLS-DA analysis evaluation for each group. **a**) MosqEVs, **b**) HepEVs, **c**) FibEVs and **d**) SynEVs.

Following the multivariate analyses, a ROC curve analysis was performed to evaluate the classification performance of the sPLS-DA model and to validate the robustness of spectral discrimination among EV types. The ROC curves quantify model accuracy by plotting the true positive rate (sensitivity) against the false positive rate (1–specificity) across multiple decision thresholds, while the AUC provides an overall measure of discriminative ability, with values close to 1.0 indicating excellent classification and values near 0.5 representing random performance. The resulting ROC curves demonstrated high predictive performance for all biological EV classes, with AUC values ranging from 0.977 to 0.994 for MosqEVs, HepEVs and FibEV groups (Fig. 6). These results confirm the model’s high capacity to accurately distinguish the biochemical profiles of EVs derived from distinct cellular sources. The SynEV group initially exhibited an inverted AUC value (0.002), which reflects a reversal in the polarity of the prediction score used for ROC computation rather than a failure of class discrimination. An AUC < 0.5 indicates anti-correlated ranking of the assigned positive class; therefore, reversing the direction of the prediction score (equivalent to calculating AUC corrected = 1 − AUC) yielded a corrected AUC of approximately 0.998. This correction resolves the sign convention without altering the underlying separation achieved by the model. The high classification performance observed for SynEVs is consistent with their controlled extrusion-based preparation from defined lipid mixtures, which results in reduced within-group heterogeneity and a more uniform spectral profile compared to naturally secreted EV populations. In contrast, biological EV preparations inherently exhibit compositional and subpopulation variability, which may slightly reduce ROC-derived metrics despite clear group separation. Furthermore, the predictive accuracy achieved through 100-fold cross-validation was 93.2% for HepEVs, 88.0% for FibEVs, 86.9% for MosqEVs, and 93.6% for SynEVs (**Figure S4**), underscoring the reproducibility and robustness of the classification model. The sparsity constraint applied in sPLS-DA, limiting the model to a subset of discriminative features, further reduces the likelihood of overfitting and supports the biological relevance of the selected spectral variables.

## Discussion

The present study demonstrates, for the first time, the application of ATR-FTIR spectroscopy to the comparative characterization of EVs derived from both vector (*Aedes albopictus* C6/36) and host (hepatocyte and fibroblasts) cellular origins as well as comparison with synthetically engineered EVs. This work establishes a new analytical perspective by correlating infrared spectral fingerprints of EVs across biological systems that represent two essential sides of the arboviral transmission cycle. To our knowledge, this is also the first report describing the ATR-FTIR spectral profile of mosquito-derived EVs providing novel insights into their biochemical composition and potential functional implications in viral dissemination.

Previous studies have shown that mosquito-derived EVs can encapsulate viral RNA and proteins, facilitating viral transfer between cells and modulating infection efficiency^20–22^. The distinctive IR spectra observed for MosqEVs, especially the elevated absorbance in lipid-associated regions, align with a distinct membrane composition of insect cells, which is substantially more symmetrical and biophysically “softer” than mammalian cells and display much lower levels of sterols due to their inability to synthesize cholesterol *de novo*. A proportionally higher abundance of phospholipids likely contributes substantially due to stronger lipid signatures^23,24^. In contrast, the mammalian EVs displayed spectra enriched in protein-associated amide I and II bands, reflecting the metabolic complexity and signaling functions of their parental cells. HepEVs derived EVs exhibited higher protein and nucleic acid content, consistent with high metabolic activity of hepatocytes^25^, whereas FibEVs presented intermediate features.

These spectral variations reflect the underlying biochemical heterogeneity among EV types and indicate a link between molecular composition, cellular origin, and biological role. The ATR-FTIR-derived biochemical fingerprints thus provide a rapid, label-free means of inferring EV function and potential infectious state, offering complementary insight to traditional molecular approaches.

Importantly, the strong classification performance of PCA, CAP, sPLS-DA, and ROC models in differentiating EV groups demonstrates that ATR-FTIR can sensitively capture subtle compositional differences linked to infection-related functions. Similar studies have used ATR-FTIR to discriminate cancerous versus healthy exosomes^9,13,26^, but this is the first to extend such spectral differentiation to mosquito-derived EVs and to propose its use for host-vector comparative analyses.

Rapid identification of EVs (and viruses) derived from specific organs is highly interesting for liquid biopsy approaches to e.g. assess organ damage or – infection in complex samples such as plasma. The present study here serves as an initial step towards analysis of extracellular vesicles and related viral particles directly within complex biological matrices, using their intrinsic vibrational fingerprints as organ- and origin-specific spectral markers. By establishing that ATR-FTIR can discriminate EVs from distinct producer cell types, this study provides a methodological foundation for developing rapid, label-free liquid-biopsy tools capable of identifying the tissue or host source of nanoscale vesicles or viruses, supporting diagnostic, toxicological, and infection-monitoring applications and builds upon existing literature focusing on difference vesicle sources and analysis pipelines (**Table ****S1**).

ATR-FTIR offers several advantages over conventional EV characterization methods, including minimal sample preparation, no need for labeling or costly reagents, and the capacity to analyze multiple biochemical components simultaneously^12,27^. Such features make it ideal for vector surveillance programs and for ensuring the reproducibility and purity of EV preparations in laboratory settings. Nevertheless, some methodological limitations must be considered, such as the influence of sample heterogeneity, the thickness of the deposited layer on the ATR crystal, and the need for confirmatory proteomic or lipidomic validation.

In perspective, this pioneering approach provides a foundation for future studies exploring EVs produced under viral infection. Extending ATR-FTIR analyses to in vitro Zika- or Dengue-virus infected cells could help identify infection-specific spectral markers and biochemical alterations, supporting its development as a rapid, field-deployable diagnostic platform for infection screening in complex biological matrices. Integrating ATR-FTIR with portable spectrometers and machine learning algorithms could further enable real-time classification and epidemiological monitoring. Ultimately, creating a comprehensive spectral database of EVs from diverse biological origins and infection states will strengthen standardization and expand the diagnostic utility of this technique. In this context, our study introduces ATR-FTIR spectroscopy as a novel and powerful tool for comparative EV analysis across host–vector systems, establishing its potential as a rapid, label-free method for infection surveillance and exosome quality assessment.

## Supplementary Information

Below is the link to the electronic supplementary material.

Supplementary Material 1

## Data Availability

Data for this article are available at OPARU (Open Access Repositorium der Universität Ulm und Technischen Hochshule UI) through this link: https:/doi.org/10.18725/OPARU-58920.
