# Correction: Spectroscopic fingerprinting of extracellular vesicles from diverse cellular origins byATR-FTIR for vibrational biomarkers of vector–host interactions

**DOI:** 10.1038/s41598-026-47422-9

**Published:** 2026-04-16

**Authors:** Emine Billur Sevinis Ozbulut, Kenta Hoshino, Yoshitomo Furushima, Yuki Yoshida, Takashi Yamamoto, Hanna Reßin, Shreyans Chatterjee, Jan Münch, Boris Mizaikoff, Rüdiger M. Groß, Lorena Diaz de Leon Martinez

**Affiliations:** 1 Toray Automotive Center Europe, Toray Industries Europe GmbH, Am Gfild 6, Neufahrn bei Freising, 85375 Neu-Isenburg, Germany; 2 Toray Research Center Inc, Shiga, 520-8567 Japan; 3 Institute of Analytical and Bioanalytical Chemistry, Ulm University, Albert- Einstein-Allee 11, 89081 Ulm, Germany; 4 Institute of Molecular Virology, Ulm University Medical Center, Meyerhofstrasse 1, 89081 Ulm, Germany

Correction to: *Scientific Reports* 10.1038/s41598-026-44338-2, published online 16 March 2026

The original version of this Article contained an error in the Author List and the Affiliation list. Specifically, the name of the author Jan Münch, which was incorrectly given as Jan Munch.

Additionally, Affiliation 4 was erroneously given as when “Kent, Ulm University Medical Center, Meyerhofstrasse 1, 89081, Ulm, Germany”. The correct Affiliation is “Institute of Molecular Virology, Ulm University Medical Center, Meyerhofstrasse 1, 89081 Ulm, Germany”. As a result Affiliation 5, which read: “Institute of Molecular Virology, Ulm University Medical Center, Meyerhofstrasse 1, 89081 Ulm, Germany” was removed, due to its redundancy.

The original Article has been corrected.

