# Supplementary material for: Spectroscopic fingerprinting of extracellular vesicles from diverse cellular origins by ATR-FTIR for vibrational biomarkers of vector–host interactions
Source: Sci Rep. 2026 Mar 16;16:9195. doi: 10.1038/s41598-026-44338-2 (PMC12996455; doi:10.1038/s41598-026-44338-2)
Supplement: Supplementary file 1 — Supplementary Material 1 [file 41598_2026_44338_MOESM1_ESM.docx]

**Supplementary Material**

**Table S1.** Comparative study of EVs through ATR-FTIR

| **Study (Year)** | **Sample / Context** | **Aim (What ATR-FTIR was used to show)** | **Chemometric / ML methods reported** | **Key takeaway** | **Reference** |
| --- | --- | --- | --- | --- | --- |
| **This study (2025)** | EVs from cell culture related to vector-host interactions | Demonstrate vector-borne, vector-host interaction-related EVs spectral differences | PCA, CAP, sPLS-DA, ROC and band analysis | ATR-FTIR provides non labelled reproducible EV fingerprinting and detects compositional changes with EVs from distinct biological origins | N/A |
| Mihály et al. (2017) | EVs from cell culture | Demonstrate feasibility of fast EV screening and compositional readouts directly from ATR-FTIR | PCA, second-derivative band analysis | ATR-FTIR provides simple, reproducible EV fingerprinting and detects compositional shifts | ^1^ |
| Szentirmai et al. (2020) | RBC-derived EVs | Reagent-free total protein quantification of intact EVs via amide-I integration | Calibration/linear regression against protein standards | Accurate, preparation-free protein quantification from EV ATR-FTIR | ^2^ |
| Romanò et al. (2020) | HT-29 cancer cell–derived exosomes | Automated classification of exosomes produced under different culture conditions | PCA + supervised models (e.g., PLS-DA/SVM) | Mid-IR fingerprints enable automated exosome classification | ^3^ |
| Stępień et al. (2021) | Melanoma lines (exosomes vs ectosomes) | Show molecular differences between EV subtypes (lipid vs protein content) | PCA, ratio metrics (lipid/protein), supervised discrimination | Ectosomes show higher lipid:protein than exosomes; robust subtype separation | ^4^ |
| Ramos-García et al. (2021) | Human milk exosomes | Routine QC of exosome isolations; link ATR-FTIR with lipidomics | PCA / PLS-DA (multimodal pipeline) | ATR-FTIR yields rapid descriptors for QC and lipid/protein estimation | ^5^ |
| Zlotogorski-Hurvitz et al. (2019) | Salivary exosomes (oral cancer vs healthy) | Diagnostic discrimination via exosome FTIR spectra | PCA, Artificial Neural Networks | Salivary exosome spectra accurately separate oral cancer from controls | ^6^ |
| Paolini et al. (2020) | EV subpopulations of different sizes/origins | Fingerprint EV subpopulations and assign source | PCA, band-target analysis | FTIR quickly fingerprints EV subtypes; supports grading/assignment | ^7^ |
| Di Santo et al. (2022) | Serum-derived EVs (cancer vs control) | Pilot liquid biopsy using EV FTIR + ML | PCA, supervised ML (reported) | EV FTIR distinguishes cancer vs non-cancer in serum samples | ^8^ |
| Praja et al. (2022) | Serum, exosomes, HDL (immunosenescence) | Discriminate elderly with high vs low pathogenic CD4⁺ T cells using exosome spectra | PCA, PLS-DA, RF/SVM/NN | Exosome ATR-FTIR carries strongest discriminatory signal (vs serum/HDL) | ^9^ |
| Martins et al. (2024) | Blood-derived EVs (Alzheimer’s disease) | Identify bdEV spectral signatures with AD discriminatory potential | Second-derivative peak areas, multivariate analyses | Differences in carbohydrate/NA, amide, and lipid regions separate AD vs control | ^10^ |
| Rajavel et al. (2025) | Blood plasma-derived EVs (Duchenne muscular dystrophy) | Identify DMD-specific EV signatures with discriminatory potential | PCA, RF | Differences in protein and lipid profiles in DMD patients. | ^11^ |

**Table S1. References**

1. Mihály J, Deák R, Szigyártó IC, Bóta A, Beke-Somfai T, Varga Z. Characterization of extracellular vesicles by IR spectroscopy: Fast and simple classification based on amide and CH stretching vibrations. *Biochimica et biophysica acta Biomembranes* 2017; **1859**(3): 459-66.

2. Szentirmai V, Wacha A, Németh C, et al. Reagent-free total protein quantification of intact extracellular vesicles by attenuated total reflection Fourier transform infrared (ATR-FTIR) spectroscopy. *Analytical and bioanalytical chemistry* 2020; **412**(19): 4619-28.

3. Romanò S, Di Giacinto F, Primiano A, et al. Fourier Transform Infrared Spectroscopy as a useful tool for the automated classification of cancer cell-derived exosomes obtained under different culture conditions. *Analytica chimica acta* 2020; **1140**: 219-27.

4. Stępień E, Kamińska A, Surman M, Karbowska D, Wróbel A, Przybyło M. Fourier-Transform InfraRed (FT-IR) spectroscopy to show alterations in molecular composition of EV subpopulations from melanoma cell lines in different malignancy. *Biochemistry and biophysics reports* 2021; **25**: 100888.

5. Ramos-Garcia V, Ten-Doménech I, Moreno-Giménez A, et al. ATR-FTIR spectroscopy for the routine quality control of exosome isolations. *Chemometrics and Intelligent Laboratory Systems* 2021; **217**: 104401.

6. Zlotogorski-Hurvitz A, Dekel BZ, Malonek D, Yahalom R, Vered M. FTIR-based spectrum of salivary exosomes coupled with computational-aided discriminating analysis in the diagnosis of oral cancer. *Journal of cancer research and clinical oncology* 2019; **145**(3): 685-94.

7. Paolini L, Federici S, Consoli G, et al. Fourier-transform Infrared (FT-IR) spectroscopy fingerprints subpopulations of extracellular vesicles of different sizes and cellular origin. *Journal of extracellular vesicles* 2020; **9**(1): 1741174.

8. Di Santo R, Vaccaro M, Romanò S, et al. Machine Learning-Assisted FTIR Analysis of Circulating Extracellular Vesicles for Cancer Liquid Biopsy. *Journal of Personalized Medicine* 2022; **12**(6): 949.

9. Praja RK, Wongwattanakul M, Tippayawat P, et al. Attenuated Total Reflectance-Fourier Transform Infrared (ATR-FTIR) Spectroscopy Discriminates the Elderly with a Low and High Percentage of Pathogenic CD4+ T Cells. *Cells* 2022; **11**(3).

10. Soares Martins T, Ferreira M, Magalhães S, et al. FTIR Spectroscopy and Blood-Derived Extracellular Vesicles Duo in Alzheimer's Disease. *Journal of Alzheimer's disease : JAD* 2024; **98**(3): 1157-67.

11. Rajavel A, Essakipillai N, Anbazhagan R, Ramakrishnan J, Venkataraman V, Natesan Sella R. Molecular profiling of blood plasma-derived extracellular vesicles derived from Duchenne muscular dystrophy patients through integration of FTIR spectroscopy and machine learning reveals disease signatures. *Spectrochimica Acta Part A: Molecular and Biomolecular Spectroscopy* 2025; **326**: 125236.

**
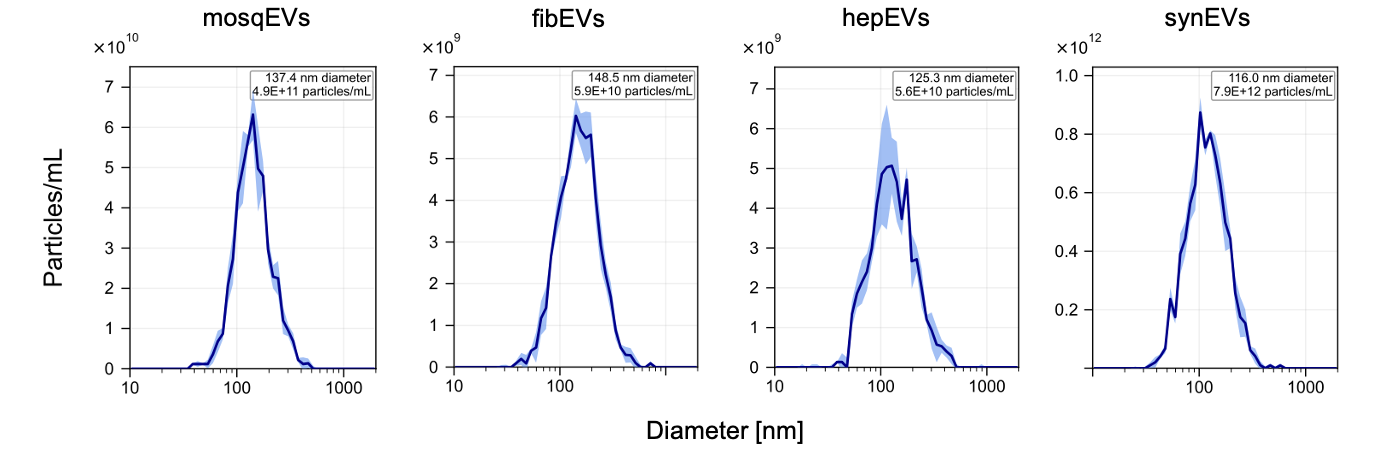
**

**Figure S1**. Size distributions measured for different EV preparations using nanoparticle tracking analysis (NTA). Shown is the median (line) and SEM (shaded area) of 3 separate measurements.


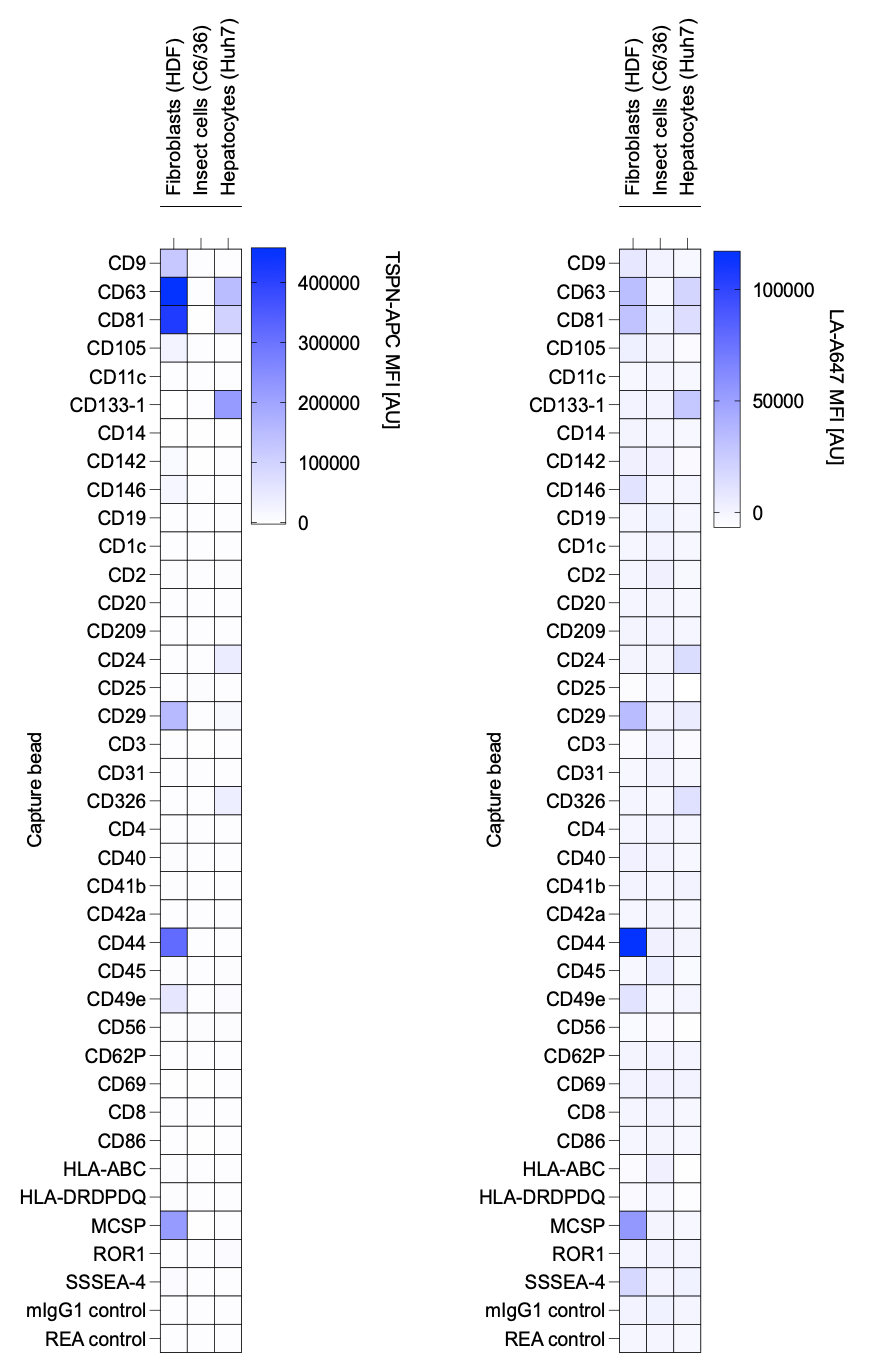


**Figure S2:** Surface marker analysis of purified EVs using bead-assisted flow cytometry (MACSPlex IO). EVs are incubated with capture beads binding indicated EV marker proteins, and EVs bound to these beads are then detected using an anti-tetraspanin (TSPN) antibody cocktail (targeting CD9, CD63, CD81) or Lactadherin (targeting PS); each detected as mean fluorescent intensity per capture bead using flow cytometry. Per analysis (TSPN and LA), 10^9^ particles based on NTA measurements were applied. Note that insect cell-derived EVs were included but did not yield any signals, as they do not contain any human proteins bound by the capture beads. Measured in duplicates, mean values are shown.


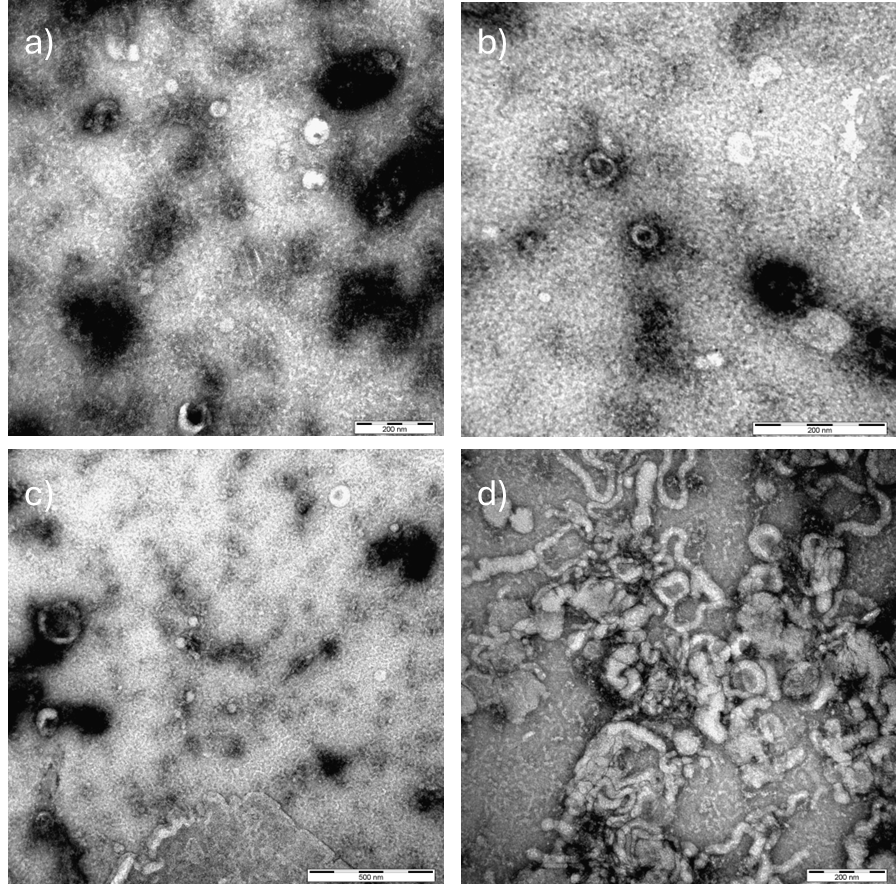


**Figure S3.** TEM analysis for each one of the EVs groups. a) MosqEVs; b) HepEVs; c) FibEVs; and d) SynEVs.


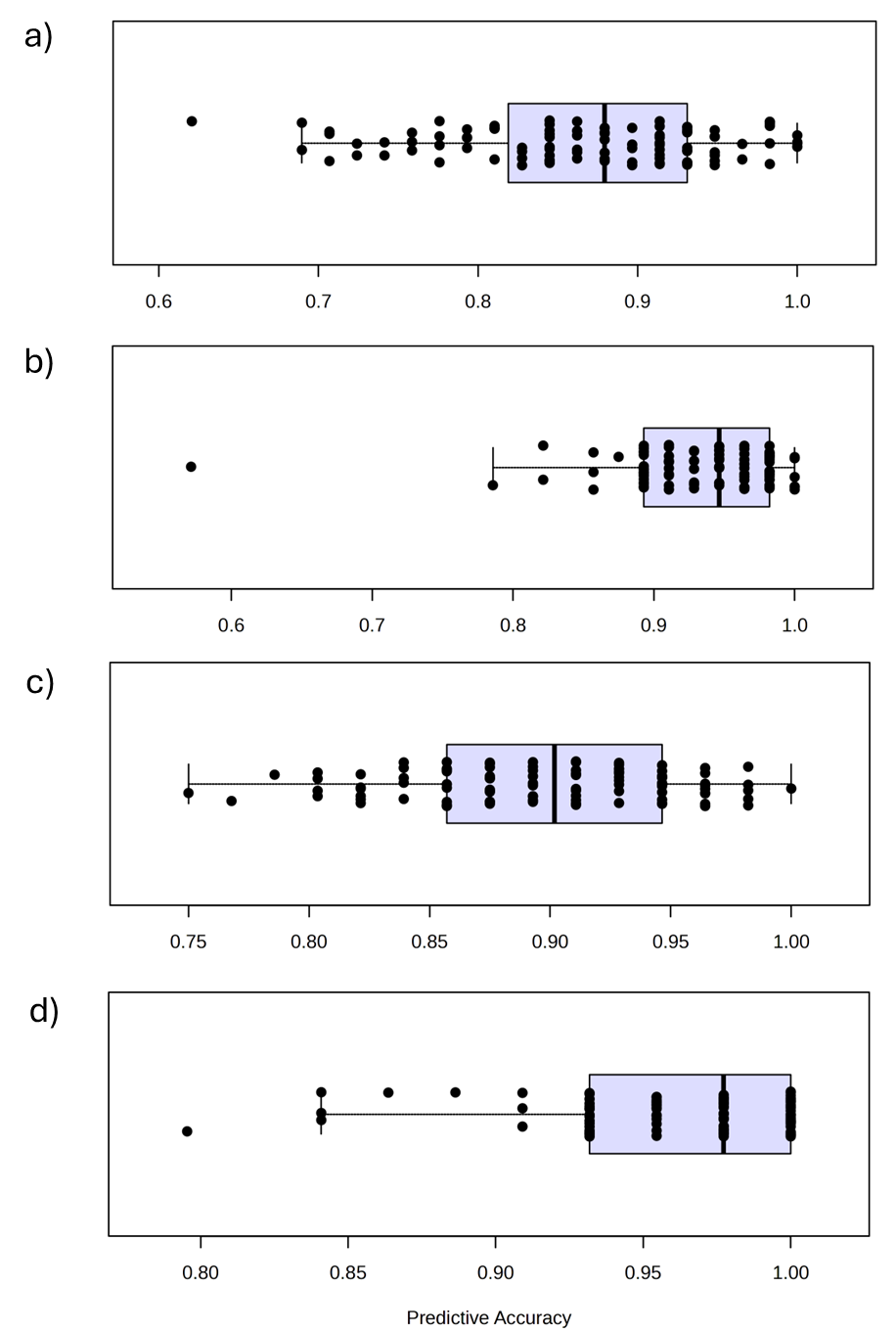


**Figure S4.** Predictive Accuracy Achieved Through 100-Fold Cross-validation. a) MosqEVs; b) HepEVs (EV57); c) FibEVs; and d) SynEVs.


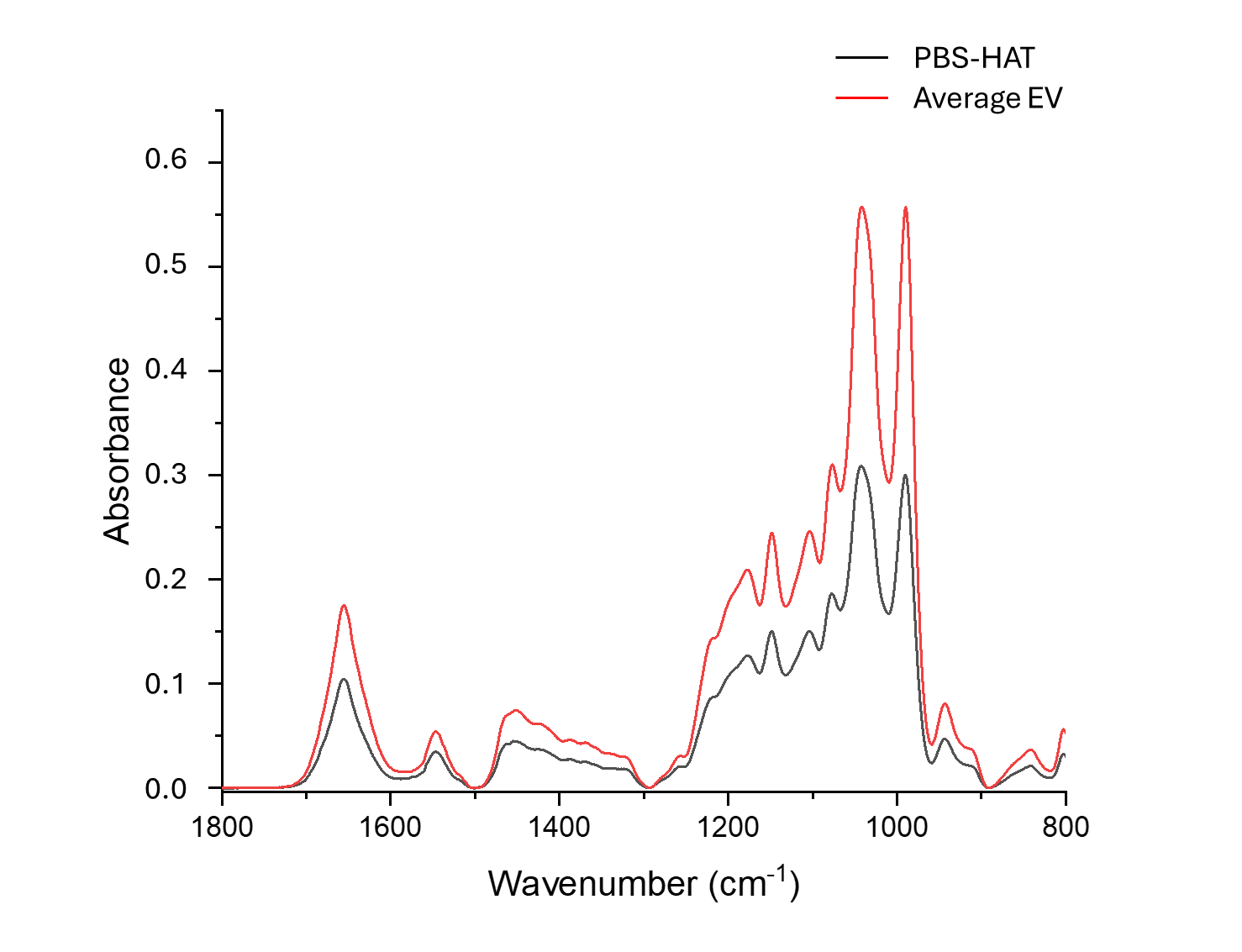


**Figure S5.** Representative ATR-FTIR spectra of PBS-HAT buffer alone compared with a normalized EV sample, demonstrating negligible background contribution from the buffer in the analyzed spectral region.
